# Ruxolitinib: Long-Term Management of Patients with Myelofibrosis and Future Directions in the Treatment of Myeloproliferative Neoplasms

**DOI:** 10.1007/s11899-014-0229-y

**Published:** 2014-08-22

**Authors:** A. Yacoub, O. Odenike, S. Verstovsek

**Affiliations:** 1 Department of Hematology and Oncology, University of Kansas Medical Center, 3901 Rainbow Boulevard, Kansas City, KS 66160 USA; 2 Section of Hematology/Oncology, University of Chicago and Comprehensive Cancer Center, 5841 S. Maryland Avenue, MC 2115, Chicago, IL 60637 USA; 3 Clinical Research Center for Myeloproliferative Neoplasia, Department of Leukemia, MD Anderson Cancer Center, 1515 Holcombe Blvd., Suite 428, Houston, 77030 TX USA

**Keywords:** Allogeneic stem cell transplantation, COMFORT-I, COMFORT-II, JAK inhibitor, Myelofibrosis, Myeloproliferative neoplasm, Polycythemia vera, Ruxolitinib, Splenomegaly

## Abstract

Considerable clinical experience regarding the long-term efficacy and safety of ruxolitinib has been gathered since the drug was approved in the USA for patients with intermediate or high-risk myelofibrosis (MF) in November 2011. Findings from the pivotal phase 3 COMFORT studies showed that ruxolitinib-associated reductions in MF-related splenomegaly and symptom burden occur rapidly and in the majority of patients. Two- and 3-year follow-up data further suggest that the benefits of ruxolitinib are durable and associated with a survival advantage compared with conventional therapies. However, careful management of treatment-related thrombocytopenia and anemia with dose modifications and supportive care is critical to allow chronic therapy. Based on preliminary evidence, ruxolitinib also allows spleen size and symptom reduction before allogeneic stem cell transplantation without negative effect on engraftment or outcomes. In recent studies, ruxolitinib provided effective management of hematologic parameters and symptoms in patients with polycythemia vera refractory to or intolerant of hydroxyurea.

## Introduction

Myelofibrosis (MF) is a highly heterogeneous, chronic, *BCR-ABL1*-negative myeloproliferative neoplasm (MPN) associated with progressive bone marrow fibrosis, extramedullary hematopoiesis, excessive production of inflammatory cytokines, and shortened survival [1–3]. In addition to arising de novo as primary MF (PMF) [4], the disease also may result from myelofibrotic transformation of other *BCR-ABL1*-negative MPNs, i.e., of polycythemia vera (PV) to post-PV MF or essential thrombocythemia (ET) to post-ET MF [5]. MF primarily affects elderly patients [6, 7], with an estimated annual incidence of two to three cases per 100,000 persons in the USA [8]. Based on the presence of specific prognostic factors, patients are classified as low, intermediate-1, intermediate-2, or high risk for early death [6, 9, 10]. Patients diagnosed with low-risk MF may survive for 15 years or longer, whereas the median life expectancy for intermediate-2 and high-risk patients is only 3 years and <2 years, respectively [9].

The clinical presentation of MF varies considerably, but most patients have some degree of spleen enlargement and many have spleen-related as well as constitutional symptoms. In advanced disease, palpable spleen length is often >10 cm [7], and debilitating spleen-related and constitutional symptoms are a major source of poor health-related quality of life (QoL) [11, 12]. Another common disease manifestation is anemia, which affects approximately 50 % of patients with PMF [7].

The pathobiology of MF and *BCR-ABL1*-negative MPNs is complex [13]. Although the vast majority of patients with MPNs have three types of essentially mutually exclusive somatic mutations, i.e., *JAK2*, thrombopoietin receptor gene (*MPL*), or calreticulin gene (*CALR*) mutations [13–15], some may have rare mutations such as *LNK* mutations [16]. Additional pathogenetic complexity can result from the presence of various mutations in epigenetic modifiers, some of which have a negative impact on patients’ prognoses [17–20]. However, irrespective of the precise mutational status, MPNs share distinct gene expression signatures that lead to overactivation of the JAK-STAT signaling pathway [21•], providing a compelling rationale for the therapeutic targeting of this pathway in patients with MPNs across different mutational backgrounds [22].

The JAK1/JAK2 inhibitor ruxolitinib to date remains the only pharmacotherapy in MF that has been approved by the US Food and Drug Administration (FDA). The results of two randomized controlled phase 3 studies, COMFORT-I and COMFORT-II, showed that ruxolitinib rapidly reduced splenomegaly and improved MF-related symptoms and QoL measures in patients with advanced MF compared with placebo or best available therapy (BAT), respectively [23, 24]. Although the COMFORT studies recruited only patients with intermediate-2 or high-risk MF and platelet counts ≥100 × 10^9^/L, other studies confirmed that symptom and spleen responses with ruxolitinib occur across risk categories, including intermediate-1 [25], and in patients with low platelet counts [26]. More recently, ruxolitinib also demonstrated clinical benefits in phase 2 and 3 studies of patients with advanced PV refractory to or intolerant of hydroxyurea [27, 28].

In this review, we discuss the current and future role of ruxolitinib in the management of patients with MPNs, including its efficacy and safety in the long-term treatment of MF and PV, its efficacy in the treatment of MF-associated complications, and its use as pretreatment for allogeneic hematopoietic stem cell transplantation (alloHSCT). In addition, we provide a brief overview of emerging therapies, including combinations with ruxolitinib and JAK inhibitors in late clinical development.

## Ruxolitinib in the Management of Patients with MF

An important finding from the COMFORT studies was that ruxolitinib provided at least some clinical benefit for the vast majority of patients who received treatment [23, 24]. In COMFORT-I, all but 5 of 155 patients in the ruxolitinib arm experienced a spleen volume reduction at week 24 [23], and in COMFORT-II, 97 % of patients randomized to ruxolitinib who had post-baseline data experienced a spleen size reduction at any time during the study [29••]. Further analysis of COMFORT-I data showed that ruxolitinib was effective across subgroups defined at baseline by MPN etiology, age, risk status, *JAK2*V617F mutation status, or platelet count, or by the presence or absence of anemia or marked splenomegaly [30]. Symptom and QoL improvement, which occurred in the majority of ruxolitinib-treated patients in the COMFORT studies, was observed for a large variety of different MF-related symptoms and parameters defined by various symptom and QoL assessment instruments, including abdominal discomfort, pain, appetite loss, fatigue, night sweats, pruritus, global health status/QoL, and physical, role, social, and emotional functioning [31, 32].

### Long-Term Efficacy and Safety in the COMFORT Studies

#### Durability of Spleen Volume Reductions and Symptom Improvement

**Fig. 1:**
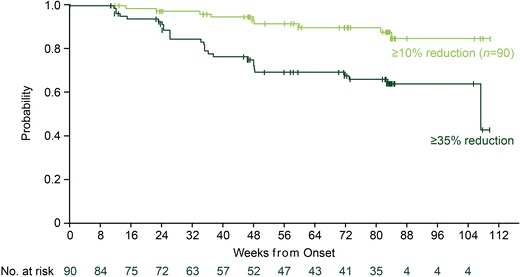
Durability of spleen volume reduction in COMFORT-I . Kaplan-Meier curve of durability of spleen volume reduction. In patients maintaining a ≥35 % reduction in spleen volume (*dark green line*), duration of response was defined as the time from the first 35 % reduction to less than 35 % reduction and 25 % increase from nadir. Among patients achieving a 35 % reduction in spleen volume, most patients maintained a ≥10 % reduction from baseline (*light green line*), with duration defined as the time from the first 35 % reduction to less than 10 % reduction from baseline [33]

Recent 2- and 3-year follow-up data from the COMFORT trials demonstrated the durability of ruxolitinib-mediated spleen size reductions and QoL benefits in patients who remain on therapy [29••, 33, 34]. In COMFORT-I, patients randomized to ruxolitinib and remaining on therapy had a mean percentage change from baseline in spleen volume of −31.6 % at week 24, −34.9 % at week 96, and −34.1 % at week 144 [33, 34]. Importantly, 100 (64.5 %) and 77 (49.7 %) of the 155 patients randomized to ruxolitinib were still on treatment at the time of the 2- and 3-year analysis, respectively [33, 34]. Similarly, 45 % (66 of 146) of the patients randomized to ruxolitinib in COMFORT-II remained on treatment at the time of the 3-year analysis, and those who achieved a ≥35 % reduction in spleen size (51 %) had a 50 % probability of maintaining this level of improvement at week 144 [29••]. In COMFORT-I, 59 % of patients randomized to ruxolitinib achieved a ≥35 % spleen volume reduction at some time during the study [34], and those patients had a >80 % probability to still have a ≥10 % reduction at the median 2-year follow-up (Fig. 1) [33]. Spleen volume reductions of as small as 10 % are clinically meaningful because of their association with effective control of symptoms and significant improvement in global health status/QoL [31]. Long-term follow-up of QoL in COMFORT-I showed that gains obtained during the first 24 weeks, including reduction in fatigue levels and increases in global health status/QoL and role and physical functioning, were generally maintained during long-term follow-up [33].

Although few patients show primary clinical resistance to ruxolitinib (lack of response), others may develop secondary clinical resistance (loss of response) after initially responding to ruxolitinib. A recent analysis in 41 patients found that four and 12 of 16 patients with clinical resistance had primary resistance (defined by the authors as a spleen volume reduction of <10 %) and secondary resistance, respectively [35]. Clinical resistance appeared not to be associated with new mutations in the JAK2 kinase drug-binding domain, but was significantly associated with the absence of *JAK2*, *MPL*, *TET2*, and *SRSF2* mutations (*P* = 0.003), and was more common in high-risk MF, post-ET MF, and patients with initially smaller spleen responses [35]. In two patients with MF, response to ruxolitinib was restored after brief withdrawal [36], suggesting that in some patients, secondary clinical resistance may be overcome with treatment interruptions. The effects of retreatment with ruxolitinib in patients with MF who interrupted treatment due to loss of response and/or adverse events will be investigated in a planned phase 2 study (NCT02091752).

#### Long-Term Safety and Management of Treatment-Related Cytopenias

In the COMFORT studies, starting doses of 15 and 20 mg twice daily (BID) were used, depending on whether the platelet count at baseline was between 100 and 200 × 10^9^/L or >200 × 10^9^/L. The most common adverse effects of ruxolitinib were dose-dependent cytopenias caused by decreases in platelet counts and temporary decreases in hemoglobin levels, particularly during the first 3 months of therapy [23, 24, 37]. These adverse effects are an expected consequence of the drug’s mechanism of action. However, thrombocytopenia and anemia were generally managed successfully with dose adjustments and/or brief treatment interruptions and red blood cell transfusions (for anemia). As a result, cytopenias rarely led to treatment discontinuation [23, 24]. Results of a COMFORT-I post hoc analysis suggest that baseline platelet counts <150 × 10^9^/L and baseline hemoglobin <10 g/dL may be clinically useful indicators of a likely need for dose adjustments or intervention for anemia, respectively, early in the course of therapy [38]. Timely and effective dose management is critical in keeping patients on therapy and avoiding unnecessary treatment interruptions and discontinuations. Experience from COMFORT-I and other studies further suggests that ruxolitinib at titrated doses of approximately 10 mg BID may be a suitable maintenance therapy for most patients, including those with low platelet counts at baseline [26, 37, 39, 40]. In COMFORT-I, most patients attained average daily doses (from week 21 to week 24) of 10 mg BID or higher, and 10 mg BID was associated with clinically meaningful reductions in spleen size and symptom burden [37]. In a single-center study of 42 patients, a dose escalation approach with a starting dose of 5 mg BID was effective and appeared to be associated with better hematologic tolerability than the standard dosing regimen used in the COMFORT studies [39]. This approach also has been shown to allow effective treatment of patients with MF and baseline platelet counts between 50 and 100 × 10^9^/L [26].

The primary results of the COMFORT studies indicated that ruxolitinib was generally not associated with severe non-hematologic adverse effects [23, 24], and neither trial reported unexpected safety concerns or increasing rates of adverse events over a 3-year follow-up period [29••, 33, 34]. However, in clinical practice, isolated cases of serious opportunistic infections have been reported [41–46], and there is evidence that ruxolitinib may have immunosuppressive properties [47–49]. Heightened vigilance is required in the treatment of patients with a compromised immune system due to MF or comorbidities.

The favorable safety record of ruxolitinib over a substantial period of clinical experience is noteworthy because of the recent safety-related termination of clinical development of two JAK2 inhibitors. XL019 was terminated after all patients developed central and/or peripheral neuropathy in clinical phase 1 [50]. Fedratinib was terminated despite its efficacy in a placebo-controlled phase 3 study [51] after its use was linked to cases of Wernicke’s encephalopathy [52], a serious neurologic condition. To date, there have been no cases of Wernicke’s encephalopathy with ruxolitinib therapy.

### Effect on Survival and Natural Disease History

Although survival analyses in the COMFORT trials have been complicated by provisions in the study design that allowed patients in the control groups to cross over to ruxolitinib under prespecified conditions, various survival estimates conducted at 1, 2, and 3 years of follow-up based on the intent-to-treat populations consistently yielded hazard ratios (HRs) that suggested a 30 to 50 % survival advantage in favor of the ruxolitinib arms (Table 1 [23, 24, 29••, 33, 34, 53]). Notably, the COMFORT-II 3-year follow-up data provided compelling evidence of a survival advantage for the ruxolitinib versus the BAT arm (Table 1) [29••]. This survival advantage was independent of the presence of prognostically detrimental mutations [54]. Pooled survival data from the COMFORT studies showed a 35 % reduction in the risk of death with ruxolitinib versus placebo or BAT (HR, 0.65; 95 % CI, 0.46–0.90; *P* = 0.01) [55].

**Table 1:** Overall survival in the COMFORT trials at 1, 2, and 3 years of follow-up

COMFORT-I			COMFORT-II		
RUX (*n* = 155) vs. PBO (*n* = 154)			RUX (*n* = 146) vs. BAT (*n* = 73)		
Median follow-up	HR (95 % CI)	*P* value*	Median follow-up	HR (95 % CI)	*P* value*
1 year (51 weeks) [23]	0.50 (0.25–0.98)	0.04	1 year (52 weeks) [24]	0.70 (0.20–2.49)	
2 years (102 weeks) [33]	0.58 (0.36–0.95)	0.03	2 years (112 weeks) [53]	0.51 (0.27–0.99)	0.041
3 years (149 weeks) [34]	0.69 (0.46–1.03)	0.067	3 years (151 weeks) [29••]	0.48 (0.28–0.85)	0.009

*P values not adjusted for multiple comparisons

*BAT* best available therapy, *CI* confidence interval, *HR* hazard ratio, *PBO* placebo, *RUX* ruxolitinib

Passamonti et al. [56] compared survival from the time of diagnosis between patients with PMF who received ruxolitinib in COMFORT-II [24] and matched patients with PMF who participated in the Dynamic International Prognostic Scoring System (DIPSS) study [10]. The DIPSS study was conducted before the advent of JAK inhibitor therapy, and patients in this study received conventional therapy [10]. The results of the comparative analysis suggested a 39 % reduction in the risk of death with ruxolitinib versus conventional therapy, with median survival times of 5 years (95 % CI, 2.9–7.8) for ruxolitinib-treated patients and 3.5 years (95 % CI, 3.0–3.9) for the DIPSS cohort from the time of diagnosis [56].

Collectively, the available survival data suggest that ruxolitinib may alter the natural history of MF; however, the basis for this effect remains a matter of debate. Elevation of specific cytokines [57], presence of specific mutations [17–19], bone marrow fibrosis grade [58, 59], splenomegaly [55, 60], and comorbidities [59] all may have prognostic implications in addition to the parameters in established scoring systems. Ruxolitinib has been shown to modify some of these factors, including splenomegaly, symptoms and symptom-related cytokine levels, and cachexia-related weight loss and hypocholesterolemia [23, 24, 61], but in general does not cause rapid or major changes in bone marrow histomorphology and generally has no major effect on mutant allele burden [24, 29••]. Thus, the life-prolonging effect of ruxolitinib may be primarily a consequence of patients’ improvement in overall clinical status. However, a complete resolution of bone marrow fibrosis has been documented in two patients with post-PV MF after 168 weeks (39 months) [62] and 17 months of ruxolitinib therapy [63]. In addition, a preliminary report showed that long-term therapy with ruxolitinib for up to 5 years halted or reversed bone marrow fibrosis progression in some patients with MF compared with a matched control group that received BAT (not including JAK inhibitors) [64]. The observed reductions in bone marrow fibrosis grade were accompanied by evidence of some improvement in the bone marrow inflammatory stromal reaction and megakaryocyte morphology [65].

### Effect on Clinical Manifestations Other Than Splenomegaly and Constitutional Symptom Burden

Case reports and results from clinical studies indicate that ruxolitinib can mitigate clinical manifestations and complications of MF besides splenomegaly and constitutional symptoms. For example, individual patients with MF who developed hepatomegaly after splenectomy achieved liver size reductions of 50 to 68 % and concomitant improvement of hepatomegaly-related symptoms [66]. In addition, normalization of blood counts has been observed in patients who had marked leukocytosis and thrombocytosis but no clinically significant spleen enlargement [66].

In a study in 15 patients with intermediate- or high-risk MF and pulmonary hypertension, treatment with ruxolitinib was associated with statistically (*P* < 0.05, compared with pretreatment values) and clinically significant improvements in hematologic and cardiac parameters [67]. These improvements were accompanied by significant reductions (*P* ≤ 0.05) in IL-4, IL-6, IL-8, and TNF-α plasma levels [67]. Preliminary results of an investigator-initiated multicenter phase 2 study of ruxolitinib in patients with MPN-associated splanchnic vein thrombosis suggest that ruxolitinib may reduce spleen and liver parenchymal stiffness (as assessed by FibroScan) and cardiac output, consistent with improvement of the splanchnic and systemic circulation [68].

### Ruxolitinib Before Allogeneic Stem Cell Transplantation

AlloHSCT remains the only potentially curable treatment for MF, but it also remains associated with high risks of mortality [69, 70]. Age, constitutional symptoms, and massive splenomegaly are considered risk factors for poor outcomes of alloHSCT [70, 71]. A number of small retrospective studies have evaluated the effects of ruxolitinib pretreatment on outcomes of alloHSCT. In a study at a single institution, 14 patients received ruxolitinib therapy before alloHSCT for 2 to 12 months [72]. This resulted in a 41 % median decrease in palpable spleen length in 9 of 11 patients with splenomegaly and a median reduction in symptom scores of 52.5 % in 11 of the 14 patients. Engraftment occurred in 13 patients (93 %). After a median follow-up of 9 months, overall survival, event-free survival, and treatment-related mortality rates were 79, 64, and 7 %, respectively.

In a German multicenter study in 22 patients, ruxolitinib treatment before alloHSCT reduced spleen size and constitutional symptoms in the majority of patients, and the estimated 1-year overall survival and disease-free survival rates were 81 and 76 %, respectively. No adverse effects of ruxolitinib withdrawal at the start of conditioning and no negative impact of treatment on engraftment were observed [73•]. Interestingly, a significant difference in overall survival rates (*P* = 0.02) between patients who showed spleen responses with ruxolitinib therapy (12/12) and those who did not (6/10) was noted. Overall, 27 % of the patients had acute graft-versus-host disease (GvHD) grade 3 or 4, and one patient died of GvHD. Analysis of T cell population at day 100 after transplantation suggested that ruxolitinib had no negative impact on immune reconstitution [73•]. A recent study further provided evidence that ruxolitinib can be effective in the treatment of steroid-refractory GvHD after alloHSCT by suppressing the production of pro-inflammatory cytokines [74].

In a French study in 11 patients with MF, treatment with ruxolitinib before alloHSCT reduced spleen size, was well tolerated, and was associated with and excellent engraftment rate. After a median of 339 days since the initiation of ruxolitinib therapy, survival was 80 % and 6 patients (54 %) were in complete remission [75]. However, preliminary results of an ongoing phase 2 study (JAK-ALLO, NCT01795677) of ruxolitinib before alloHSCT in 22 patients with MF revealed unexpected, potentially treatment-related, serious adverse events after ruxolitinib withdrawal and initiation of preparative chemotherapy regimen, namely tumor lysis syndrome (*n* = 3) and cardiogenic shock (*n* = 3) [76]. Another phase 2 study evaluating ruxolitinib as pretreatment for reduced-intensity alloHSCT in MF is currently recruiting patients (NCT01790295).

## Future Perspectives

### Ruxolitinib-Based Combination Therapy for MF

Although ruxolitinib therapy represents a major advance in the management of MF, its benefit has limitations, including lack of improvement in cytopenias and other manifestations of the marrow failure state associated with advanced MF. Available preclinical and early-phase clinical data suggest that agents with complementary or synergistic activity may include anti-fibrotic agents such as anti-lysyl oxidase-like-2 antibodies [77–79] and PRM-151 (recombinant human pentraxin-2) [80], inhibitors of epigenetic dysregulation such as histone deacetylase inhibitors [79, 81–83], other kinase inhibitors targeting the JAK-STAT pathway [84, 85], and hedgehog inhibitor LDE225 [86]. Ongoing studies of ruxolitinib-based combination therapy are listed in Table 2. Of note, a recent phase 1b dose-finding study of ruxolitinib and panobinostat in patients with MF revealed a tolerable safety profile for this combination and encouraging spleen size reductions [87].

**Table 2:** Ongoing trials of ruxolitinib combination therapy in MF

Clinical trial number	Phase	Combination	Patients	Sponsor
NCT01693601 (PRIME)	1/2	Ruxolitinib + panobinostat	Intermediate-2 or high-risk MF, platelet count ≥75 × 10^9^/L	Mount Sinai School of Medicine
NCT01433445	1b	Ruxolitinib + panobinostat	MF, platelet count >100 × 10^9^/L	Novartis
NCT01732445	2	Ruxolitinib + danazol	Intermediate- or high-risk MF, platelet count ≥50 × 10^9^/L, and anemia (hemoglobin <10 g/dL or transfusion dependent)	Mayo Clinic
NCT01644110	1/2	Ruxolitinib + pomalidomide	MF with anemia (hemoglobin <10 g/dL or transfusion dependent)	University of Ulm
NCT01375140	2	Ruxolitinib + lenalidomide (with or without prednisone)	Intermediate- or high-risk MF, platelet count ≥100 × 10^9^/L	MD Anderson Cancer Center
NCT01369498	2	Ruxolitinib + GS-6624	Intermediate- or high-risk MF	Gilead Sciences
NCT01981850	2	Ruxolitinib + PRM-151	Intermediate- or high-risk MF	Promedior, Inc.
NCT01787552	1/2	Ruxolitinib + LDE225	Intermediate- or high-risk MF, platelet count ≥75 × 10^9^/L	Novartis
NCT01730248	1b	Ruxolitinib + BKM120	Intermediate- or high-risk MF, platelet count ≥75 × 10^9^/L	Novartis

*MF* myelofibrosis

Although immunomodulatory drugs may be used to treat cytopenias, they are generally associated with dose-limiting toxicities and modest activity in MF as single therapy [88, 89]. However, they may be useful in combination with JAK inhibitor therapy. A number of combination studies of ruxolitinib with anti-anemia drugs are currently underway, including a phase 2 study of ruxolitinib plus danazol in patients with advanced MF and anemia, a phase 2 study of ruxolitinib plus lenalidomide in patients with MF, and a phase 1b/2 study of ruxolitinib plus pomalidomide in patients with MF and anemia (Table 2).

### Emerging Therapies for MF

Two JAK inhibitors now in phase 3 clinical development, pacritinib and momelotinib, have shown promise in improving the management of patients with MF and low platelet counts or anemia. Furthermore, in an ongoing investigator-sponsored single-center study, a number of patients treated with the telomerase inhibitor imetelstat achieved complete morphologic and/or molecular remission [90]. However, in March 2014, the imetelstat study was placed on partial clinical hold by the FDA because of suspected treatment-related hepatotoxicity [91].

#### Pacritinib

Combined data from phase 2 studies showed that pacritinib, a JAK2/FLT3 inhibitor, reduced spleen volume by ≥35 % in 43 % of evaluable patients with MF and platelet counts ≤100 × 10^9^/L [92]. Analysis of changes in Common Terminology Criteria (CTC) grade for hemoglobin levels and platelet counts revealed improvement by 1 grade in 16 and 18 % and worsening by 1 grade in 28 and 30 % of patients, respectively. Of 57 patients who had platelet counts of ≤100 × 10^9^/L and were subject of an integrated safety analysis across phase 1 and phase 2 studies, 46 % had no CTC grade change from baseline in hemoglobin level or platelet count [92]. These findings suggest that pacritinib may have less of a myelosuppressive effect than ruxolitinib, particularly on platelets. A phase 3 study is currently evaluating the efficacy and safety of pacritinib versus BAT (including ruxolitinib) in patients with MF and thrombocytopenia (NCT02055781).

#### Momelotinib (CYT387)

In a phase 1/2 study of the JAK1/JAK2 inhibitor momelotinib in patients with advanced MF, 39 % of evaluable patients who completed nine or more treatment cycles of 28 days had a spleen response, and 46 to 100 % of patients experienced ≥50 % improvement in individual symptom scores. In addition, 68 % of previously red blood cell transfusion–dependent patients became transfusion independent, while thrombocytopenia (29 %), neutropenia (5 %), and lipase elevation (4 %) were the most common grade 3 or 4 adverse events related to therapy [93]. The mitigating effect on anemia reported for momelotinib is unique among JAK inhibitors and has been proposed to result from treatment-related effects on specific cytokines, possibly including IL-1 receptor antagonist (IL-1RA) and IL-1β [94]. A randomized BAT-controlled (not including ruxolitinib) phase 3 study evaluating the efficacy and safety of momelotinib in patients with MF and anemia or thrombocytopenia who were treated with ruxolitinib is planned (NCT02101268). In addition, a comparative phase 3 study of momelotinib and ruxolitinib in patients with MF is ongoing (NCT01969838).

### Ruxolitinib for PV and Other *BCR-ABL1*-Negative Hematologic Neoplasms

In a phase 2 study of ruxolitinib in 34 patients with advanced PV refractory to or intolerant of hydroxyurea, treatment resulted in rapid and durable clinical benefits [27]. A hematocrit level <45 % without phlebotomy was achieved in 97 % of patients by week 24, with a 61 % probability of maintaining this response at week 144. Of patients with palpable splenomegaly at baseline, 63 % had a non-palpable spleen at week 144. In addition, ruxolitinib therapy resulted in clinically meaningful improvement of symptoms (pruritus, night sweats, and bone pain) and sustained reductions in white blood cell counts in most patients. Grade ≥3 thrombocytopenia and grade ≥3 anemia occurred in three patients each (five patients overall) and were managed with dose modifications and/or treatment interruptions [27]. A preliminary report from the RESPONSE trial (NCT01243944), a randomized open-label phase 3 study in 222 phlebotomy-dependent patients with PV and splenomegaly and resistance or intolerance to hydroxyurea, indicated that 21 % of patients randomized to ruxolitinib 10 mg BID (*n* = 110) compared with 1 % of patients randomized to BAT (*n* = 112) achieved both hematocrit control (defined as no more than one phlebotomy in the first 8 weeks post randomization and no eligibility for phlebotomy based on hematocrit levels from weeks 8 to 32) and a ≥35 % reduction in spleen volume at week 32 (*P* < 0.0001) [28]. In the ruxolitinib and BAT arms, respectively, 60 versus 20 % of patients achieved hematocrit control, 38 versus 1 % had a ≥35 % reduction in spleen volume, 49 versus 5 % had a ≥50 % reduction in symptom burden, and 24 versus 9 % had a complete hematologic response. One patient treated with ruxolitinib and 6 patients who received BAT had thromboembolic events during the first 32 weeks. Grade 3 or 4 anemia occurred in 1.8 and 0 % of patients in the ruxolitinib and BAT arms, respectively; the corresponding percentages for grade 3 or 4 thrombocythemia were 5.5 and 3.6 %, respectively [28]. Another phase 3 study is currently investigating the efficacy and safety of pegylated interferon-α2a versus hydroxyurea in patients with high-risk PV or ET (NCT01259856).

Case reports suggest that ruxolitinib therapy also may benefit patients with other chronic leukemias characterized by overactive JAK-STAT signaling. Hematologic and symptom improvement with ruxolitinib treatment was recently reported for two patients with Chuvash polycythemia [95]. In addition, a patient with chronic neutrophilic leukemia (CNL) and a point mutation (T618I) in the colony-stimulating factor 3 receptor gene *CSF3R* experienced rapid and marked reductions in white blood cell and absolute neutrophil counts [96]. This is consistent with preclinical data showing that CNL and atypical chronic myeloid leukemia associated with oncogenic *CSF3R* mutations are sensitive to JAK inhibition [96].

## Conclusion

Ruxolitinib provides rapid and durable clinical benefits in MF that may extend beyond spleen size reduction, symptomatic mitigation, and QoL improvement, including reducing hepatomegaly or improving splanchnic thrombosis or pulmonary hypertension. In addition, ruxolitinib therapy has been shown to be effective in the treatment of patients with PV who are refractory to or intolerant of hydroxyurea, and treatment results for individual cases raise the possibility that ruxolitinib may benefit specific patients with Chuvash polycythemia or CNL.

Ruxolitinib therapy in MF has been associated with a survival advantage compared with conventional therapies. Ruxolitinib appears to alter the natural history of MF primarily by improving overall health status and/or modifying risk factors, but in some patients, long-term therapy also may halt or even reverse the progression of bone marrow fibrosis.

Combination therapies with ruxolitinib and emerging therapies are currently in development and may further improve outcomes and/or address remaining clinical needs such as anemia. Preliminary evidence also suggests that ruxolitinib can be beneficial as pretreatment for alloHSCT, but further investigation in controlled trials is necessary to thoroughly evaluate its effect on post-transplantation outcomes.
